# Somatic instability of the expanded GAA repeats in Friedreich’s ataxia

**DOI:** 10.1371/journal.pone.0189990

**Published:** 2017-12-19

**Authors:** Ashlee Long, Jill S. Napierala, Urszula Polak, Lauren Hauser, Arnulf H. Koeppen, David R. Lynch, Marek Napierala

**Affiliations:** 1 Department of Biochemistry and Molecular Genetics, University of Alabama at Birmingham, Birmingham, Alabama, United States of America; 2 Department of Molecular Carcinogenesis, Center for Cancer Epigenetics, University of Texas MD Anderson Cancer Center, Smithville, Texas, United States of America; 3 Children’s Hospital of Philadelphia, Philadelphia, Pennsylvania, United States of America; 4 VA Medical Center, Albany, New York, United States of America; King’s College London, UNITED KINGDOM

## Abstract

Friedreich’s ataxia (FRDA) is a genetic neurodegenerative disorder caused by transcriptional silencing of the *frataxin* gene (*FXN*) due to expansions of GAA repeats in intron 1. FRDA manifests with multiple symptoms, which may include ataxia, cardiomyopathy and diabetes mellitus. Expanded GAA tracts are genetically unstable, exhibiting both expansions and contractions. GAA length correlates with severity of FRDA symptoms and inversely with age of onset. Thus, tissue-specific somatic instability of long GAA repeats may be implicated in the development of symptoms and disease progression. Herein, we determined the extent of somatic instability of the GAA repeats in heart, cerebral cortex, spinal cord, cerebellar cortex, and pancreatic tissues from 15 FRDA patients. Results demonstrate differences in the lengths of the expanded GAAs among different tissues, with significantly longer GAA tracts detected in heart and pancreas than in other tissues. The expansion bias detected in heart and pancreas may contribute to disease onset and progression, making the mechanism of somatic instability an important target for therapy. Additionally, we detected significant differences in GAA tract lengths between lymphocytes and fibroblast pairs derived from 16 FRDA patients, with longer GAA tracts present in the lymphocytes. This result urges caution in direct comparisons of data obtained in these frequently used FRDA models. Furthermore, we conducted a longitudinal analysis of the GAA repeat length in lymphocytes collected over a span of 7–9 years and demonstrated progressive expansions of the GAAs with maximum gain of approximately 9 repeats per year. Continuous GAA expansions throughout the patient’s lifespan, as observed in FRDA lymphocytes, should be considered in clinical trial designs and data interpretation.

## Background

Friedreich’s ataxia (FRDA), the most common hereditary ataxia, is a multi-organ disorder with an approximate prevalence of 1 in 50,000 [1]. The majority of patients (> 95%) are homozygous for a guanine-adenine-adenine (GAA) repeat expansion in the first intron of the *frataxin* (*FXN*) gene. In FRDA patients, the expanded GAA repeat tracts are associated with locus-specific chromatin changes leading to transcriptional silencing [2–4] of the *FXN* gene and consequent deficiency of the mitochondrial protein, frataxin [1, 5, 6].

FRDA has a variable clinical presentation with onset typically between ages 5–15 and the vast majority of patients exhibiting symptoms by age 25 [1, 7, 8]. The first symptoms of FRDA often include ataxia, sensory impairment, and scoliosis. The heart is also affected, resulting in cardiomyopathy and cardiac dysfunction, with 60% of FRDA deaths reflecting cardiac dysfunction [7, 9, 10]. Approximately 10–30% of patients develop dysfunction of pancreatic beta-cells, including impaired glucose tolerance and diabetes mellitus [8, 9]. The severity of symptoms in FRDA correlates with the size of the expanded GAA repeats, and likely reflects the effects of frataxin deficiency on different tissues.

GAA repeats are polymorphic in unaffected individuals, with lengths ranging from 7–22 copies [1]. The expanded GAA tracts typically contain between 200 and 900 or more repeats, with the majority of alleles containing 700–800 copies of the GAA motif [1]. In unaffected individuals GAA repeats are stable with some infrequently occurring contractions or expansions. However, GAA instability can be seen in premutation *FXN* alleles, containing 26–44 uninterrupted repeats [11–14]. Intergenerational instability is observed in the expanded GAA repeats, with a preference for contractions in the paternal line while the maternal line shows both contractions and expansions [15]. Comparative analysis of paired blood and sperm samples from FRDA patients revealed shorter repeat lengths in sperm, occasionally with the GAA tract contractions resulting in unaffected allele sizes [15].

In addition to intergenerational instability, expansions and contractions of GAA repeats in somatic cells have been detected in FRDA, with different tissues containing varying GAA lengths within the same patient [1, 13–22]. Analysis of GAA instability in dorsal root ganglion (DRG) samples from six patients showed a pronounced bias towards repeat expansions [18]. In addition, heterogeneity of the GAA repeat tract was detected in a limited number of samples from other tissues, including blood, cerebrum, cerebellum, spinal cord, brainstem, and heart [18]. *FXN* GAA repeats in the cerebellum showed an expansion bias, while the lowest number of large contractions in the repeat tract was observed in DNA isolated from a heart sample [18]. Somatic GAA instability was also detected throughout different regions of the brain, including four cerebral cortical areas, the dentate nucleus and cervical spinal cord [21]. Additionally, two case studies reported differences of GAA tract size in the sural nerve compared to lymphocytes [16, 20]. GAA length heterogeneity was also observed when comparing repeats in DNA samples isolated from paired lymphocytes and cultured fibroblasts of FRDA patients, however, no bias for expansions or contractions was noted [22]. While longitudinal studies of GAA repeat size in an FRDA cohort have not been reported, analyses of expanded GAA repeat tracts in cerebrum, cerebellum, spinal cord, heart, and pancreas of a 24-year-old patient and an 18-week fetus indicated an age-dependent increase of instability, thus suggesting an accumulation of changes in expanded GAA length throughout the lifetime [19].

Along with human studies, somatic instability has been reported in the humanized YAC transgenic YG8 and YG22 as well as the YG8sR FRDA mouse models [17, 23]. These mice harbor relatively short tracts of 190 + 82 (two tandem YAC transgene GAA sequences), 190, and **~**200 GAA repeats, respectively [17, 23]. Similar to the results obtained from patient tissues, the mouse tissues showed an expansion bias, particularly in the cerebellum and DRG, as well as an age-dependent increase in mutation load [17, 23]. Lastly, *in vitro* studies on cultured immortalized lymphoblast cell lines and induced pluripotent stem cells (iPSCs), obtained by reprogramming of FRDA fibroblasts, demonstrated somatic instability when serially passaged [24–26].

Although somatic instability in FRDA has been reported, systematic analyses of GAA heterogeneity in multiple tissues affected by frataxin deficiency in a larger cohort are lacking. Herein, we determined the somatic instability of the expanded GAA repeats in five tissues from 15 FRDA patients: heart, cerebral cortex, spinal cord, cerebellum, and pancreas. In addition, we compared GAA tract lengths in paired fibroblast and lymphocyte samples isolated from 16 FRDA patients. Finally, we have conducted a longitudinal analysis of GAA repeat tract lengths in lymphocytes based on repeated blood sampling from FRDA patients. Tissue-specific differences and variability in the number of GAA repeats may be one of the most important, but also the most difficult to ascertain, factors underlying clinical variability among FRDA patients. As demonstrated in our study, the dynamics of expanded GAA repeat length over time could have significant implications for correlating disease severity and interpretation of data from clinical trials.

## Methods

### Patient tissue and DNA samples

All research involving patient/patient tissues has been approved by the Institutional Review Boards (IRB) at The University of Alabama at Birmingham (IRB Protocols: N160923005 and N160922011) and Children’s Hospital of Philadelphia (IRB Protocol 10–007864). Autopsy specimens of heart, cerebral cortex, spinal cord, cerebellar cortex, and pancreas of 15 FRDA patients were obtained from the FRDA tissue repository maintained at the Veterans Affairs Medical Center in Albany, NY, USA. Tissue samples were divided into aliquots upon arrival and stored at -80°C. Blood and genomic DNA samples from five FRDA patients used in longitudinal analyses were obtained from The Children’s Hospital of Pennsylvania (CHOP, Philadelphia, PA). Blood was stored at 4°C until the DNA extraction. Fibroblast lines were derived from skin biopsies performed at CHOP as described in [27], with approvals from CHOP and UAB IRBs. All FRDA patient material used in this study was derived from patients carrying homozygous GAA repeat expansions. Cerebral and cerebellar cortex DNA from unaffected individuals were obtained from The Cooperative Human Tissue Network (Southern Division, University of Alabama at Birmingham) and Dr. Mark Pook (Brunel University, London, UK).

### DNA isolation

Genomic DNA from patient tissue samples was isolated using the DNeasy Blood & Tissue kit (cat. 69504; Qiagen). Genomic DNA was isolated from blood samples using the PureLink^™^ Genomic DNA Mini kit (cat. K1820-00; Invitrogen) or Quick-gDNA^™^ Blood MidiPrep (cat. D3074; Zymo Research). All isolations were conducted according to manufacturers’ recommendations. Genomic DNA concentration, purity, and quality were determined through measurement on a NanoDrop 2000c Spectrophotometer (ThermoFisher SCIENTIFIC) as well as visualization on a 0.7% agarose gel.

### PCR amplification

Amplification of GAA repeat expansions in the *FXN* gene was performed by PCR using primers GAA_F: 5’–GGCTTGAACTTCCCACACGTGTT and GAA_R: 5’–AGGACCATCATGGCCACACTT as previously described [26, 28, 29]. Amplifications were conducted in 40 or 50μL reactions containing 50-200ng of DNA template. The thermal cycler was programmed for an initial denaturation step of 3 minutes at 94°C, followed by 20 cycles of 20 seconds at 94°C, 30 seconds at 64°C, and 5 minutes at 68°C, followed by 9 cycles of 20 seconds at 94°C and 5 minutes at 68°C, with each subsequent elongation step increased by 15 seconds. A final extension step of 7 minutes at 68°C was then performed.

Amplification of GAA repeats at the 5q23 locus was performed with primers 5q23F: 5’–GTTGCATAGATAAATCAAATTCAT and 5q23R: 5’–ACTCACAGAAAGTATTATTATTCC [30, 31]. Amplifications were conducted in 25μL reactions containing 100ng of DNA template. The thermal cycler was programmed for a denaturation step of 3 minutes at 94°C, followed by 30 cycles which consisted of 30 seconds at 94°C, 30 seconds at 50°C, and 2 minutes at 72°C, with a final extension step of 2 minutes at 72°C.

PCR amplification of the intron1-exon2 region of the *FXN* gene, approximately 9kbp from the GAA repeat tract, was performed using primers In1Ex2F: 5’–AGCACTCGGTTACAGGCACT and In1Ex2R: 5’–GCCCAAAGTTCCAGATTTCC as previously described [32]. Amplifications were performed in 20μL reactions containing 50-100ng of DNA template. The thermal cycler was programmed for an initial denaturation step of 3 minutes at 95°C, followed by 35 cycles of 30 seconds at 95°C, 30 seconds at 60°C, and 30 seconds at 72°C, with a final extension step of 7 minutes at 72°C.

Reactions utilized the FailSafe PCR System with mix D (cat. FS99250; Epicentre) or JumpStart^™^ REDTaq^®^ ReadyMix^™^ Reaction Mix (cat. P0982; Millipore Sigma). The amplification products were resolved on 0.9–1% agarose gels stained with ethidium bromide. Lane analyses were performed using Image Lab 5.0 software (BioRad. The length of an expanded GAA tract was determined using the base pair size called by Image Lab 5.0, with the total GAA length calculated by subtracting the length of the sequences flanking the GAA repeats, including the length of the PCR primers, from the number of base pairs of the PCR product and dividing the difference by three: [Number of GAA repeats = (length of base pairs of a PCR product—498)/3].

### Western blot

Lysates were prepared using a RIPA buffer containing 150 mM NaCl, 1% IGEPAL^®^ CA-630, 0.5% sodium deoxycholate, 0.1% sodium dodecyl sulfate (SDS), 50mM Tris-HCl, pH 8, and a protease inhibitor cocktail (cat. P8340; Millipore Sigma). The Bradford Protein Assay Kit (cat. #500–0006; Bio-Rad) was used to determine protein concentration. Fifty micrograms of tissue protein lysate were electrophoresed on NuPAGE^™^ 4–12% Bis-Tris protein gels (cat. NP0322BOX; ThermoFisher SCIENTIFIC) and transferred onto nitrocellulose membranes (cat. 162–0112, Bio-Rad). Human frataxin was detected with the anti-frataxin polyclonal H-155 antibody (cat. sc-25820; Santa Cruz) and human voltage dependent anion channel (VDAC) was detected with the anti-VDAC polyclonal antibody (cat. #4866; Cell Signaling Technology), both used at 1:1,000 dilutions. Human heat shock protein (Hsp) 60 was detected with the anti-Hsp60 D307 antibody (cat. #4870; Cell Signaling Technology) at 1:2,000 dilution. GAPDH was detected with the anti-GAPDH antibody (cat. MAB374; Millipore) at 1:25,000 dilution. Primary antibody incubation was performed for at least 12 hours at 4°C. Sheep anti-mouse immunoglobulin (NA931V; GE Healthcare) and donkey anti-rabbit immunoglobulin (NA934V; GE Healthcare), linked to horseradish peroxidase, were used as secondary antibodies at a 1:5,000 dilution for 1 hour at room temperature. Signal was exposed using Amersham ECL Prime Western Blotting Detection Reagent (cat. RPN2232; GE Healthcare) or SuperSignal^®^ West Dura Extended Duration Substrate (cat. 34075; ThermoFisher SCIENTIFIC) and measured using Image Lab 5.0. Quantification was performed using ImageJ software (National Institutes of Health).

### Statistical analysis

Statistical analyses were done using IBM^®^ SPSS^®^ Statistics Version 24 and Excel 2016 software. A p-value less than 0.05 was considered significant for all analyses.

## Results

To determine whether somatic instability is present in the expanded GAA tracts of the *FXN* gene in FRDA patient tissues, we analyzed sizes of the repeats in heart, cerebral cortex, spinal cord, cerebellar cortex, and pancreas samples from 15 patients (Table 1). These samples represent a broad spectrum of tissues affected by frataxin deficiency in FRDA patients. Samples were obtained from six males and nine females, with a mean age of disease onset at ~12±7 years and a mean age of death at ~43±18 years (Table 1). The majority of patients died of heart-related causes (60%) and cachexia (27%). Quantitative western blot analyses demonstrated reduced frataxin levels in patient tissues compared to controls, confirming FRDA status of all samples (S1 Fig).

**Table 1:** Characterization of the FRDA patient tissues.

Patient	Sex	Age of onset (years)	Age of death (years)	Disease duration (years)	Tissues Available
M1	M	10	24	14	Cc, Sc, Cb
M2	M	8	27	19	H, Cc, Sc, Cb, P
M3	M	9	33	24	H, Cc, Sc, Cb, P
M4	M	9	37	28	H, Cc, Sc, Cb, P
M5	M	7	35	28	H, Cc, Sc, Cb, P
M6	M	16	46	30	H, Cc, Sc, Cb, P
F1	F	12	24	12	H, Cc, Sc, Cb, P
F2	F	10	47	37	H, Cc, Sc, Cb, P
F3	F	17	50	33	H, Cc, Sc, Cb, P
F4	F	5	25	20	H, Cc, Sc, Cb, P
F5	F	7	28	21	H, Cc, Sc, Cb, P
F6	F	7	55	48	H, Cc, Sc, Cb
F7	F	18	67	49	H, Cc, Sc, Cb, P
F8	F	15	69	54	H, Cc, Sc, Cb, P
F9	F	34	77	43	Cc, Sc, Cb, P
Mean ± SD	M, 6; F, 9	12.3 ± 7.2	42.9 ± 17.6	30.7 ± 13.1	
Range		5–34	24–77	12–54	

Abbreviations: F, female; M, male; SD, standard deviation; H, heart; Cc, cerebral cortex; Sc, spinal cord; Cb, cerebellar cortex; P, pancreas.

**Fig 1:**
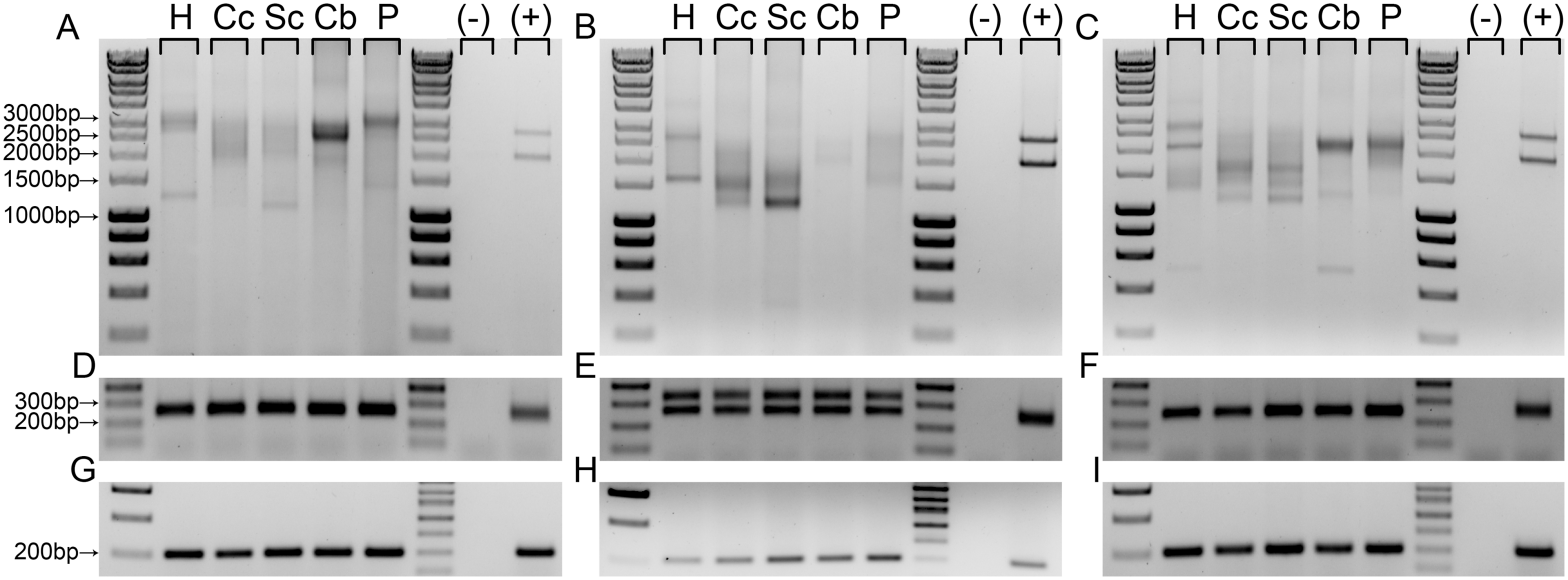
Instability analysis of the expanded GAAs in the *FXN* gene across different somatic tissues. Genomic DNA was extracted from heart (H), cerebral cortex (Cc), spinal cord (Sc), cerebellar cortex (Cb) and pancreas (P) tissues and the GAA repeats in the *FXN* locus were amplified by PCR. The results from FRDA patients (**A**) F2 (**B**) F7 and (**C**) M6 shown as examples. (-) represents no-template control and (+) represents positive control for amplification of the expanded GAAs (genomic DNA isolated from fibroblasts obtained from an unrelated FRDA patient). (**D-F**) A GAA repeat tract at the 5q23 locus was amplified by PCR using the same genomic DNA templates used for reactions shown in (**A-C**). (**G-I**) A fragment spanning intron 1—exon 2 of the *FXN* gene, downstream of the GAA tract, was also amplified using the same templates to serve as a control for genomic DNA quality.

The sizes of the GAA tracts by PCR differed across five tissues analyzed in samples from all 15 FRDA patients (13 for the heart and pancreas samples; Fig 1A–1C and S2 Fig). Typically, several bands or even a broad smear of the PCR products containing expanded GAAs are observed following agarose gel electrophoresis (Fig 1A–1C). As a positive control to ensure that the PCR conditions yielded robust amplification of expanded GAAs for each experiment, genomic DNA was isolated from an FRDA fibroblast cell line and used as a template alongside genomic DNA isolated from patient tissues. The fibroblast line used was derived form an individual unrelated to the patients from whom the tissues were obtained. Amplification of expanded GAAs using DNA obtained from the FRDA fibroblasts revealed two distinct *FXN* alleles (Fig 1A–1C, positive control lane), indicating greater somatic instability in heart, cerebral cortex, spinal cord, cerebellar cortex, and pancreas when compared to homogeneous primary cell lines derived from patients. Furthermore, amplification of a polymorphic repeat tract at locus 5q23, previously shown to contain ≥44 GAA triplets [31], revealed a lack of somatic instability between analyzed tissues (Fig 1D–1F and S2 Fig). In parallel, PCR amplification of a ~200 bp intron1-exon2 fragment of the *FXN* gene demonstrated that the quality of genomic DNA isolated from all tissues allowed for efficient amplification (Fig 1G–1I and S2 Fig).

**Fig 2:**
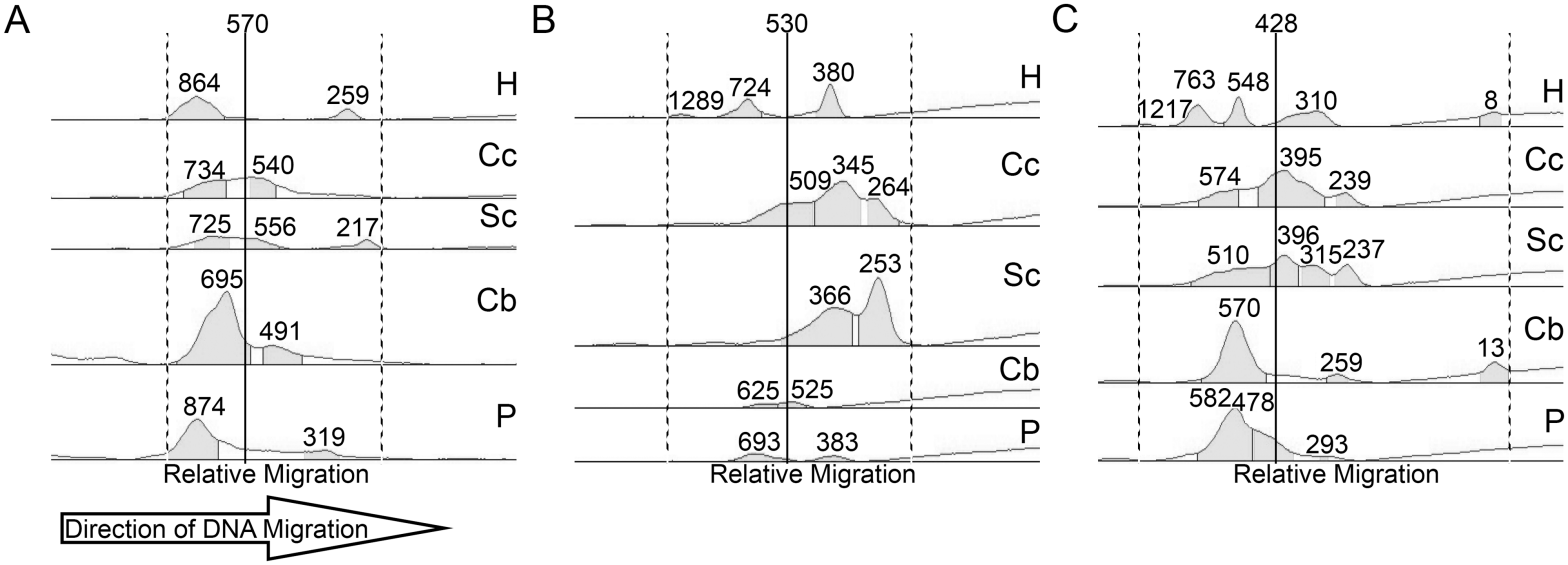
Quantitative analysis of expanded GAA repeat instability in the *FXN* gene across different tissues. The expanded GAA repeats in the *FXN* gene were amplified from genomic DNA extracted from heart (H), cerebral cortex (Cc), spinal cord (Sc), cerebellar cortex (Cb) and pancreas (P) tissues isolated from FRDA patients. The band intensity of the PCR products and repeat size are shown for FRDA patients (**A**) F2, (**B**) F7, and (**C**) M6. Solid vertical lines represent the mean of all GAA repeat sizes detected across all five tissues for each patient. Gel lanes were manually outlined and gel bands were detected via the Image Lab 5.0’s band finder set to high sensitivity. Faint bands of PCR products not detected by the software were manually identified. Band boundaries, accounting for smearing, were automatically outlined by the program with final manual adjustments to include the entire spectrum of PCR products. Multiple PCR analyses with determinations of GAA lengths were performed to demonstrate reproducibility of PCR and reliability of measurements (S4 Fig).

Next, we performed quantitative analyses of PCR products containing expanded GAAs to determine tissue-specific differences in the stability of the repeat tracts. As it was impossible to assign a precise size of each expanded allele (GAA1 and GAA2) in the majority of samples due to the high level of somatic instability, we developed an unbiased GAA length analysis protocol based on defining the minimum, maximum, and mean GAA size of the repeat tracts detected in each sample (Methods). The mean GAA repeat sizes among 15 FRDA patients and 5 different tissues were between 428 and 914 GAAs (Figs 1 and 2 and S2 and S3 Figs). The median difference between maximum and minimum GAA tract size is 726 GAAs (Figs 1 and 2 and S2 and S3 Figs).

**Fig 3:**
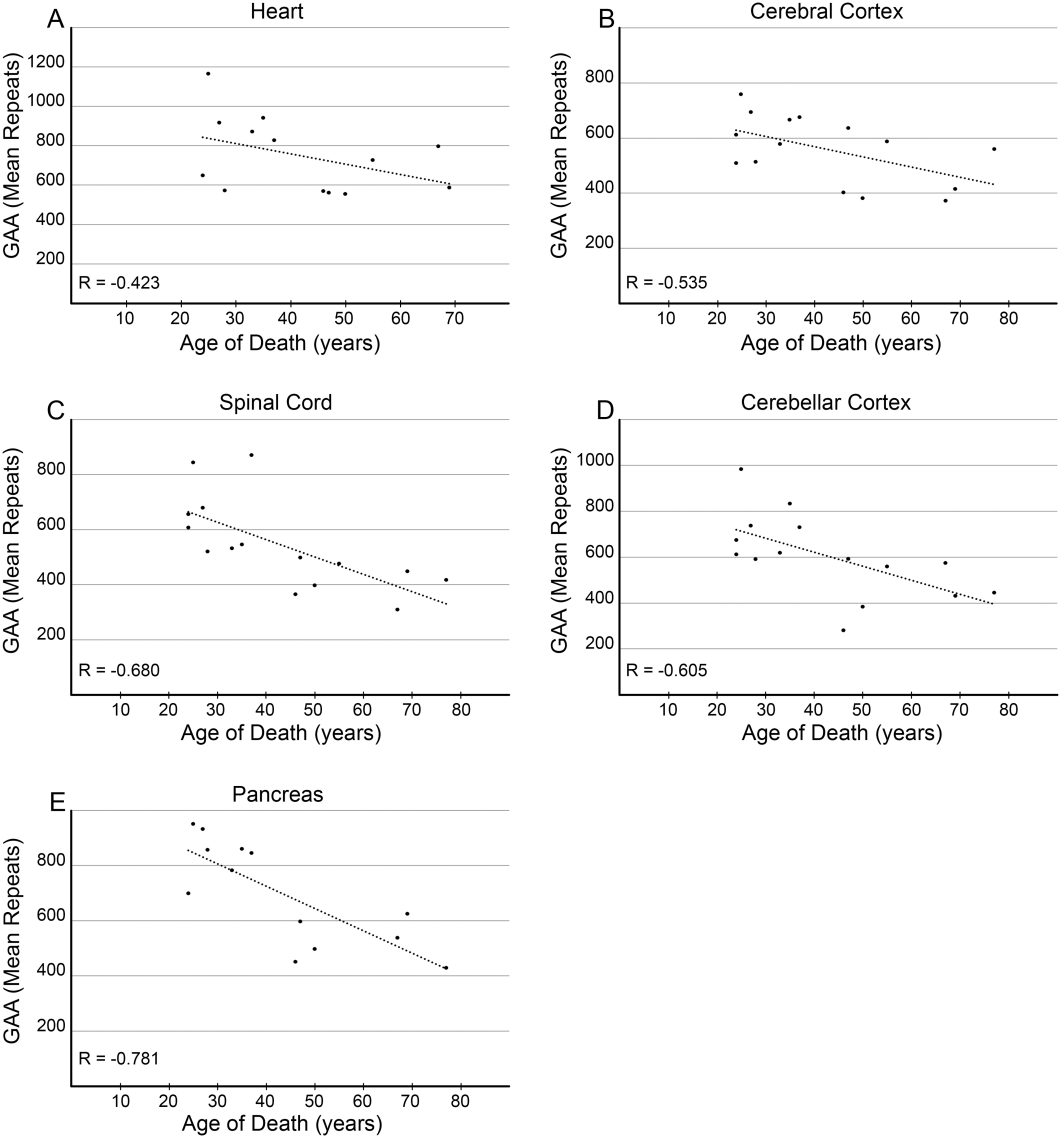
Correlative analyses between mean GAA repeat sizes and patient age of death. The mean tissue GAA tract length in FRDA patient (**A**) heart, (**B**) cerebral cortex, (**C**) spinal cord, (**D**) cerebellar cortex, and (**E**) pancreas is plotted against the patient’s age at time of death. Statistical significance was reached for spinal cord, cerebellar cortex and pancreas (p <0.05).

Interestingly, comparisons of the GAA length between 5 tissues in a repeated measures analysis revealed that the average GAA size in the heart and pancreas is significantly longer than GAAs present in cerebral cortex, spinal cord, and cerebellar cortex of the same patient (p-value <0.05; Fig 2A–2C and S3 Fig). The mean GAA sizes in the heart and pancreas were 752 (standard deviation (SD): 190) and 720 (SD: 167) GAAs compared to 614 (SD: 186), 552 (SD: 168) and 551 (SD: 131) triplet repeats in cerebellar cortex, spinal cord, and cerebral cortex, respectively. The longest mean GAA tracts were detected in either the heart or pancreas samples in 11 out of 14 patients. In contrast, the shortest mean GAA tracts were detected in either the cerebral cortex or the spinal cord tissue samples in 14 out of 15 patients. For all tissues, the number of GAA repeats correlated inversely with age of death, with Pearson’s R values ranging from -0.423 (heart) to -0.781 (pancreas). This trend reached statistical significance in spinal cord, cerebellar cortex, and pancreas (p-values <0.05; Fig 3). For comparison, we analyzed lengths of the GAA repeat tracts in cerebral cortex and cerebellar cortex samples from 3 unaffected individuals. The number of GAA repeats in all non-FRDA tissues was less than 30 and no somatic instability was observed (S5 Fig).

### Lengths of GAA tracts in paired FRDA fibroblast and lymphocyte samples

**Fig 4:**
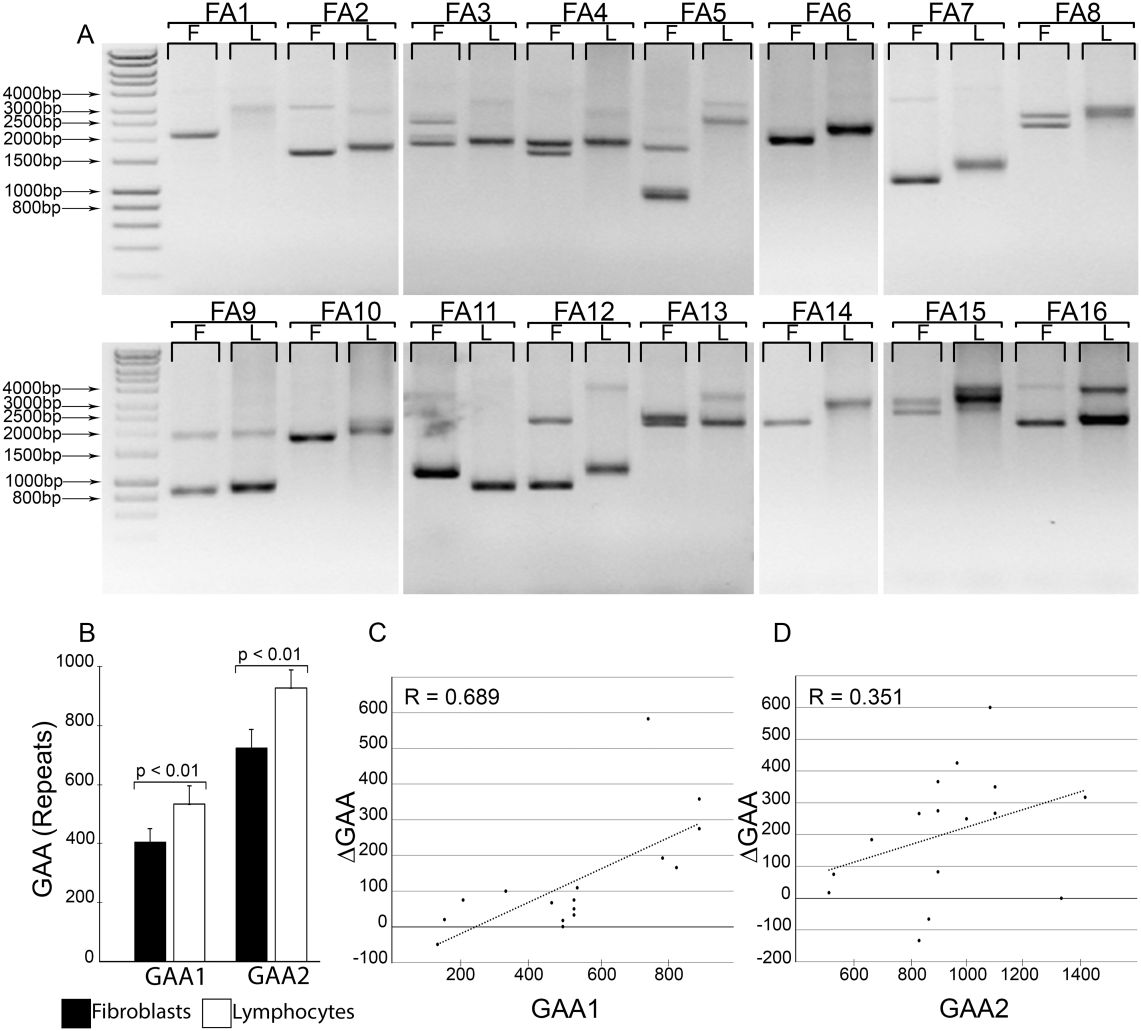
Determination of GAA repeat length in paired FRDA patient fibroblast (F) and lymphocyte (L) samples. (**A**) Agarose gel analysis of GAA repeat sizes in fibroblast/lymphocyte samples isolated from the same individual (FRDA patients FA1—FA16). (**B**) The mean sizes of the GAA1 and GAA2 alleles between all fibroblast and lymphocyte samples (n = 16) were calculated and compared. A p-value <0.05 denotes a significantly significant difference. (**C**, **D**) Correlation between the number of GAA repeats expanded in lymphocytes relative to size of the repeat tracts in fibroblasts and the number of GAA repeats in lymphocytes. The difference between the expanded GAA repeat lengths observed in lymphocyte and fibroblast samples (ΔGAA) was plotted against lymphocyte GAA sizes for each of the 16 FRDA paired samples. The analysis was performed for both alleles (**C**) GAA1 and (**D**) GAA2. The Pearson’s correlation coefficient (R) is indicated.

Patient fibroblasts and lymphocytes are commonly used in vitro models of human diseases. To determine whether the number of GAAs differ between fibroblasts and lymphocytes obtained from the same FRDA patients, we performed PCR analysis of GAA sizes in 16 paired samples (Table 2). Interestingly, in all 16 patient sample sets, longer GAA repeats were detected in lymphocytes compared to fibroblasts, in at least one allele (Fig 4A). The *FXN* gene contained larger GAA expansions in 27 out of 32 alleles (84.4%; Fig 4A) when analyzed from lymphocyte DNA. Only 3 *FXN* alleles exhibited GAA contractions in lymphocytes relative to the corresponding GAA alleles in fibroblasts (FA2, FA11 and FA16; Fig 4; Table 2), while repeat lengths of 2 alleles did not change (FA1 and FA13; Fig 4; Table 2). The median increase in repeat number detected in FRDA lymphocytes over the fibroblasts was 75 triplets for GAA1 and 258 repeats for GAA2 corresponding to 21% and 33% expansions, respectively. The maximum detected gain in GAAs reached ~600 triplet repeats (FA5; Fig 4A). The somatic expansions in FRDA lymphocytes versus fibroblasts differed significantly (p <0.01 by paired two sample for means t-test performed for both alleles; Fig 4B). In addition, a correlation exists between the number of GAA repeats gained in lymphocytes relative to size of the repeat tracts in fibroblasts and the number of GAA repeats in lymphocytes, indicating that longer GAA repeat sequences are prone to larger expansions (Fig 4C and 4D). Taken together, these data demonstrate a significantly greater and length dependent propensity for expansion of GAA repeat tracts in FRDA lymphocytes compared to fibroblasts.

**Table 2:** GAA repeat length in fibroblasts and lymphocytes of FRDA patients.

Patient	Fibroblasts:		Lymphocytes:	
	GAA1	GAA2	GAA1	GAA2
FA1	542	1333	900	1333
FA2	400	967	456	833
FA3	500	750	533	1100
FA4	433	533	542	900
FA5	167	483	750	1083
FA6	458	458	533	533
FA7	233	1100	333	1417
FA8	667	817	833	900
FA9	133	500	153	517
FA10	483	483	533	667
FA11	183	833	133	1100
FA12	133	542	208	967
FA13	500	567	500	833
FA14	625	625	900	900
FA15	600	750	792	1000
FA16	483	933	500	867

### Longitudinal analysis of GAA instability

**Fig 5:**
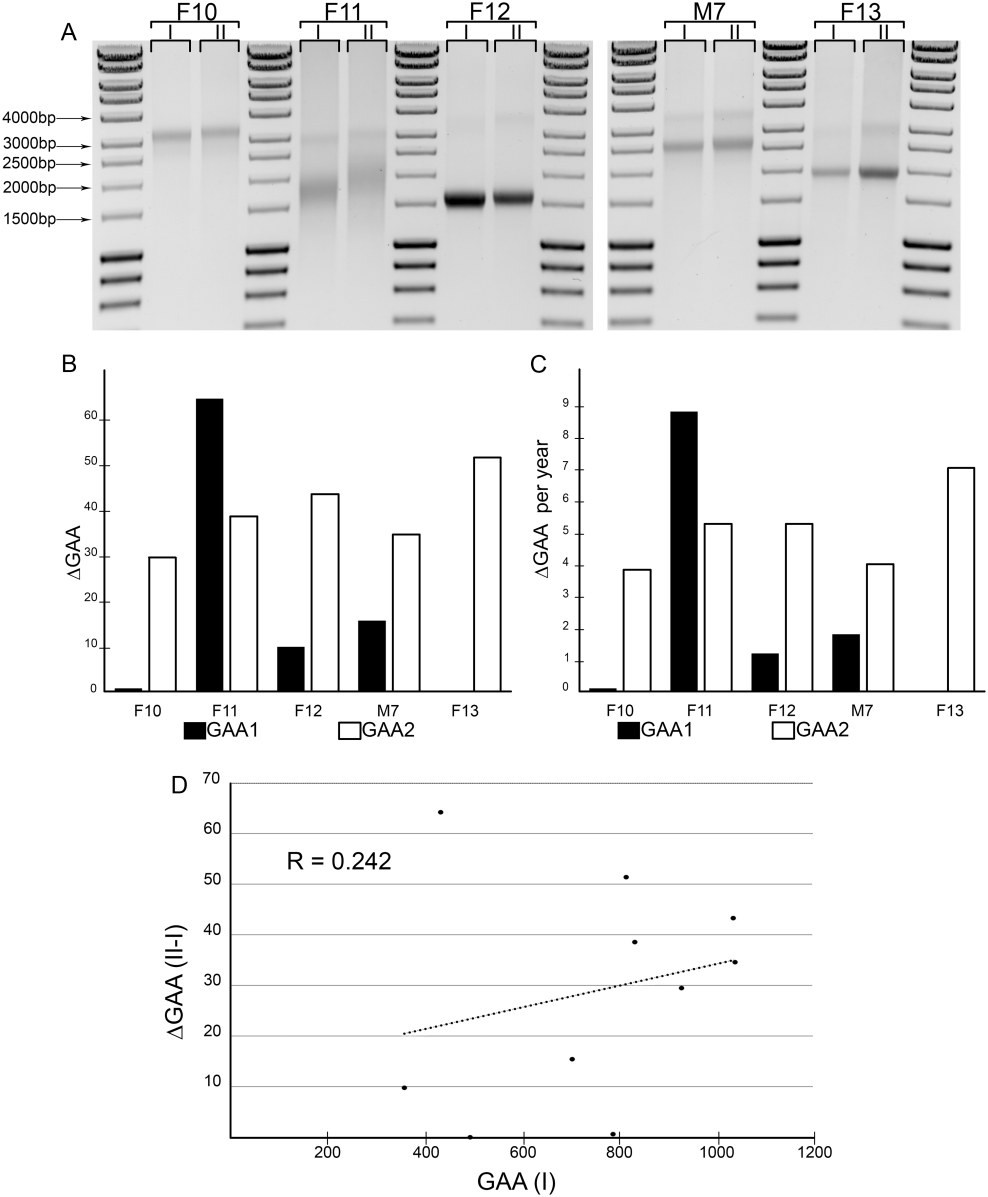
Assessing time-dependent changes of GAA tract length in FRDA lymphocytes. (**A**) The GAA repeat tract at the *FXN* locus was amplified using genomic DNA extracted from lymphocytes, which were isolated from 5 patients (F10, F11, F12, M7 and F13). Blood samples were taken at an initial timepoint (I) and a second time point 7–9 years after the initial sampling (II) (Table 3). The time-dependent changes in GAA repeat length were quantitated as (**B**) total GAA repeat gain/loss (ΔGAA) and (**C**) rate of the change (ΔGAA per year). (**D**) The Pearson’s correlation coefficient was calculated using the size of the GAA tract at time point I [GAA(I)] and the change of the number of GAAs between time points II and I [ΔGAA (II-I)]. Five pairs of samples (n = 10 alleles) were analyzed.

To determine whether lengths of the expanded GAA tracts change over time in FRDA patient blood samples, we performed a longitudinal analysis of repeat size in peripheral lymphocytes. PCR analyses of GAA length were conducted using samples collected from five patients over a span of 7–9 years (time point I—initial sample, time point II—second sample; Table 3). Differences in the number of repeats between the two time points were detected in all five patients, in at least one allele (Fig 5A). The GAA repeat sizes determined at the initial time point ranged from ~357 to ~1038 repeats (median of ~800). Interestingly, 8 out of 10 alleles exhibited an increase in size of ~10 repeats or greater and none of the analyzed alleles contracted over time (Fig 5B). The maximum detected expansion of 64 GAAs corresponds to a ~15% increase over 7.3 years (F11; Fig 5). The median expansion size over the 7–9 year range was ~32 GAAs (3.3%), indicating a yearly increase in the number of GAAs of ~4 repeats (maximum detected ~9 GAAs/year; Fig 5C). There is a direct correlation between the number of GAA repeats detected in the *FXN* gene at the time of initial sampling and the change in GAA repeat number over time, indicating a trend for longer repeat tracts to expand at a greater rate than shorter tracts (Fig 5D). Overall, these results demonstrate a bias toward GAA repeat expansion in FRDA patient lymphocytes with a propensity to expand that is dependent on the initial size of the GAA tract.

**Table 3:** Characterization of FRDA patients.

Patient	Age of Onset (years)	Interval between I and II ^a^
F10	11	7 y, 9 mo
F11	14	7 y, 4 mo
F12	20	8 y, 3 mo
M7	10	8 y, 8 mo
F13	16	7 y, 4 mo

^a^ Repeated blood samplings (I and II) were performed at the intervals indicated in the table.

## Discussion

Friedreich’s ataxia presents with considerable variability in the age of onset, symptoms, and progression. A well-established correlation exists between the number of GAA repeats in the shorter allele detected at the diagnosis and the age of onset as well as development of select symptoms, with GAA1 being a good predictor of broadly defined disease severity [33]. It has been estimated that an increase of the GAA1 tract by 100 repeats translates to ~2.3 years earlier disease onset [34]. Typically, the association between GAA number and age of onset reaches R values of 0.6–0.75 [35]. Although strong, this correlation is far from perfect and numerous “outliers” have been observed with FRDA patients lacking certain disease hallmarks or presenting with symptoms unexpectedly early based on the number of GAAs determined at diagnosis. Prior studies demonstrated that GAA lengths estimated from lymphocytes, skin fibroblasts or buccal cells may not reflect exactly the size of expanded repeat tracts in pathologically relevant tissues, such as DRG neurons or spinal cord [16, 18–22]. Although some aspects of somatic instability of the expanded GAA repeats in FRDA tissues were reported [13–22], no comprehensive or comparative analyses of GAA tract status in larger cohorts and in various tissue samples have been conducted.

In this work we addressed three separate, unanswered questions: (i) Is there a correlation between the sizes of expanded GAA tracts and tissue type, especially tissues primarily affected in the disease? (ii) Is the number of GAA repeats identical in different tissues? (iii) Do contractions or expansions of the GAA tracts accumulate over time in tissues? To answer these questions, we thoroughly characterized GAA tracts in a large number of different FRDA and control tissue samples.

Analyses of the GAA repeat region in pancreas, heart, cerebral cortex, cerebellar cortex and spinal cord isolated from 15 different FRDA patients demonstrated significant somatic mosaicism. Unexpectedly, we detected significantly longer GAA tracts in the heart and pancreas when compared to DNA samples isolated from central nervous system tissues. Considering that DNA replication, repair and transcription are three major molecular processes involved in the stimulation or prevention of repeat instability, tissue specific differences in lengths of the GAA tracts can be expected. In fact, activity of the mismatch repair (MMR) system is considered a major factor contributing to GAA repeat instability in human cells. Expansions of the GAA repeats in iPSCs reprogrammed from patient fibroblasts have been correlated with increased expression of the MMR enzymes when compared to the parental fibroblast cells [36, 37] and overexpression of MMR components stimulated GAA expansions in FRDA fibroblasts [38]. Thus, a combination of the number of replication cycles, tissue-specific transcriptional activity of the *FXN* gene and differences in the activity of various DNA repair systems could be responsible for the significantly longer GAAs detected in pancreas and heart tissues. Interestingly, quantitative Western blot analyses of frataxin expression in different tissues from FRDA patients and unaffected controls indicate a remarkably low level of frataxin in FRDA cardiac tissue relative to nervous system tissue (S1 Fig). Developmentally regulated, tissue specific cell division and replication patterns, aggravated by conditions of decreased frataxin levels, may facilitate expansion of the GAA repeats.

Transcriptome analyses conducted using FRDA mouse models demonstrated a global decrease of gene expression in frataxin deficient tissues [39]. Also, recent studies conducted in our laboratory showed that a subset of genes involved in transcription, translation and DNA repair is downregulated in FRDA cells [40]. Decreased expression of these genes may enable somatic instability of the GAAs in a tissue-specific manner. Somatic expansions that further decrease frataxin levels can in turn reinforce global transcriptome defects and perpetuate expansions of the GAA tract feeding a “somatic instability cycle”. Furthermore, difficulties of handling intracellular reactive oxygen species (ROS), characteristic of frataxin deficient cells, may stimulate DNA damage, further augmenting GAA instability, similar to the “toxic oxidation cycle” proposed to explain somatic instability of CAG repeats in Huntington’s disease (HD) [41]. Considering an important role of ROS in FRDA pathogenesis, the use of potent mitochondrial ROS scavengers inhibited somatic instability of CAG repeats and delayed phenotype development in the HD mouse model, and may also inhibit progressive somatic expansions of the GAAs in FRDA [41].

Interestingly, results of the longitudinal analyses of the GAA tract in lymphocytes, although conducted on a relatively small pool of expanded alleles, demonstrated a strong, length-dependent bias towards repeat expansions. Eight of 10 alleles analyzed increased in GAA length by up to 64 repeats over a period of 7–9 years. This result contrasts with prior in vitro studies on immortalized lymphoblast cells where a predominance for contractions, frequently large, was observed [24]. In fact, in vitro culturing of FRDA cells or model cell lines harboring expanded GAAs results in shortening of the expanded repeats with a notable exception of iPSCs where continuous expansion of the GAA tracts was observed [25, 26]. This parallel between the FRDA iPSCs and peripheral blood lymphocytes suggest that the specificity of the pluripotent/multipotent state (iPSC and hematopoietic stem cells or lymphoid progenitors) may facilitate GAA expansions. It is difficult to ascertain whether the longer GAA tracts detected in lymphocytes result from progressive expansions of the repeats in this lineage or from contractions in the development and culturing of skin fibroblasts (or both processes at the same time). Results of the longitudinal analyses described in this work strongly favor progressive expansions in lymphocytes or their progenitors. Additionally, to date we have never detected contractions during prolonged culture of FRDA primary skin fibroblasts. The inherent characteristics of the stem cells, including chromatin status, replicative properties, transcriptional and repair activity may contribute to the expansion bias observed in these cells.

Results of our longitudinal studies also indicate that comparative analyses of the GAA length between patients need to be interpreted cautiously as expanded tracts change dynamically over a patient’s lifetime. Considering the extreme case of a 15% increase over 7 years (Fig 5), substantial discrepancies in the number of GAAs can exist between a diagnosis made early in life and adulthood. Frequently, symptom correlation studies include the lengths of the GAA1 and GAA2 alleles. Furthermore, enrollment criteria for clinical trials also sometimes consider the number of GAA repeats. Therefore, periodic follow-up determinations of GAA length should be conducted. These data may also be directly beneficial to the FRDA patient community. The GAA repeat number is a frequent topic of discussion during meetings with FRDA patients, both between health professionals and patients as well as among patients themselves. FRDA patients and caregivers frequently directly associate the repeat tract size with differences in symptoms and disease progression. Thus, additional factors affecting repeat length, such as tissue-specific instability or timing of the molecular diagnosis of the disease as described in this study, may help explain imperfect relationships that sometimes exist between disease status and the number of GAAs detected in blood samples at diagnosis. It is also important to consider secondary genetic modifiers that can contribute to age of onset and disease symptoms, for example, the milder FRDA presentation observed in the Acadian population when compared to patients of other ethnic origins carrying GAA repeat tracts of similar size [22] or earlier age of onset associated with heterozygosity for p. C282Y variant in hemochromatosis (*HFE*) gene [42].

Length polymorphism and somatic instability of the expanded GAA repeat tracts in different tissues represent the first layer of complexity responsible for differences in frataxin expression that can translate to clinical variability between FRDA patients. It has been estimated that the length of the repeat tract accounts for only 36% of the variability in the age of onset with other contributing factors being genetic, epigenetic, or environmental in nature [34]. The *FXN* GAA tract evolved from G/A rich sequences of the ancient Alu elements. It is highly likely that long GAA sequences constantly evolve and change, resulting in introduction of point mutations (interruptions) into the pure GAA motifs. Examples of such interruptions have been recurrently reported in the literature but their actual detailed structure and frequency in *FXN* alleles is unknown. Disturbance of the GAA repeat purity can affect not only expression of the *FXN* gene but also can significantly influence instability of the repeat tract, especially the propensity for continuous expansions. Although technically challenging, future studies addressing the role of GAA tract interruptions in FRDA heterogeneity are necessary.

## Conclusions

Expanded GAA repeats are unstable in FRDA, and expansion bias detected in heart and pancreas is likely to contribute to symptom development and disease progression, making the mechanism of somatic instability an important target for therapy. Differences in the size of the GAA tracts between lymphocytes and fibroblasts urge caution in direct comparisons of data obtained using different model systems. Continuous GAA expansions over time as observed in FRDA lymphocytes should be considered in clinical trials design and data analyses. The kinetics of longitudinal changes in GAA repeat number and potential *cis* and *trans* acting factors affecting this process need to be further investigated.

## Supporting information

S1 Fig Quantitative western blot analyses of frataxin expression. Western blots probed for FXN were performed on (**A,D**) heart, (**B,E**) cerebral cortex, and (**C,F**) cerebellar cortex tissues. Frataxin expression values were normalized using the mitochondrial HSP60 protein. Patient F9 exhibited very low HSP60 expression in cerebellar cortex tissue, therefore GAPDH was used for normalization.
(PDF)

S2 Fig Instability of the expanded GAAs in the *FXN* gene in different tissues. Genomic DNA was extracted from heart (H), cerebral cortex (Cc), spinal cord (Sc), cerebellar cortex (Cb) and pancreas (P) tissues. Results of PCR analyses of GAA repeat length in FRDA patient tissues (top panel). Analyses of GAA repeat instability in the 5q23 locus in patient tissues (middle panel). Amplification of an intron 1—exon 2 fragment of the *FXN* gene downstream of the GAA tract as control for genomic DNA quality (bottom panel). (-) represents no-template control and (+) represents positive control (genomic DNA isolated from FRDA fibroblasts). (**A**) F1, (**B**) F4, (**C**) F5, (**D**) F3, (**E**) F6, (**F**) F8, (**G**) F9, (**H**) M1, (**I**) M2, (**J**) M3, (**K**) M5, and (**L**) M4 as described in Table 1.
(PDF)

S3 Fig Quantitative analysis of expanded GAA instability in the *FXN* gene in different tissues. The expanded GAA repeats in the *FXN* gene were amplified from genomic DNA extracted from heart (H), cerebral cortex (Cc), spinal cord (Sc), cerebellar cortex (Cb) and pancreas (P) tissues isolated from FRDA patients. The band intensity of the PCR products along with the repeat sizes are shown. Solid vertical lines represent the mean of GAA repeat sizes detected across all tissues analyzed. (**A**) F1, (**B**) F4, (**C**) F5, (**D**) F3, (**E**) F6, (**F**) F8, (**G**) F9, (**H**) M1, (**I**) M2, (**J**) M3, (**K**) M5, and (**L**) M4 as described in Table 1.
(PDF)

S4 Fig Reproducibility of PCR analyses of the expanded GAAs in different tissues. The expanded GAA repeats in the *FXN* gene were amplified from genomic DNA extracted from heart (H), cerebral cortex (Cc), spinal cord (Sc), cerebellar cortex (Cb) and pancreas (P) tissues isolated from FRDA patients in two independent reactions. Analyses of GAA repeat instability in tissues of FRDA patient F2; (**A**) experiment 1, (**B**) experiment 2 and in tissues of FRDA patient M5 (**C**) experiment 1, (**D**) experiment 2. Sizes of the individual PCR products were calculated for each tissue and experiment. No significant differences were observed between sizes of the GAA tracts determined in two independent experiments (t-test, p > 0.05).
(PDF)

S5 Fig GAA repeat tracts in the *FXN* gene of unaffected individuals show no instability. PCR analysis of the GAA repeat region in the *FXN* gene using genomic DNA extracted from the cerebral cortex (Cc) and cerebellar cortex (Cb) tissues of unaffected individuals. (-) represents no-template control and (+) represents positive control (genomic DNA isolated from control fibroblasts).
(PDF)

## Abbreviations

Cb: Cerebellar cortex
Cc: Cerebral cortex
Ctl: Control
F: Female
FRDA: Friedreich’s ataxia
FXN: Frataxin
GAA: Guanine-adenine-adenine
GAA1: Shorter guanine-adenine-adenine allele
GAA2: Longer guanine-adenine-adenine allele
GAPDH: Glyceraldehyde 3-phosphate dehydrogenase
H: Heart
HD: Huntington’s disease
HSP60: Heat shock protein 60
iPSCs: Induced pluripotent stem cells
M: Male
MMR: Mismatch repair
P: Pancreas
PCR: Polymerase chain reaction
ROS: Reactive oxygen species
Sc: Spinal cord
SD: Standard deviation
