# Supplementary figures and images for: Somatic instability of the expanded GAA repeats in Friedreich’s ataxia
Source: PLoS One. 2017 Dec 19;12(12):e0189990. doi: 10.1371/journal.pone.0189990 (PMC5736210; doi:10.1371/journal.pone.0189990)

S1 Fig. Long et al.

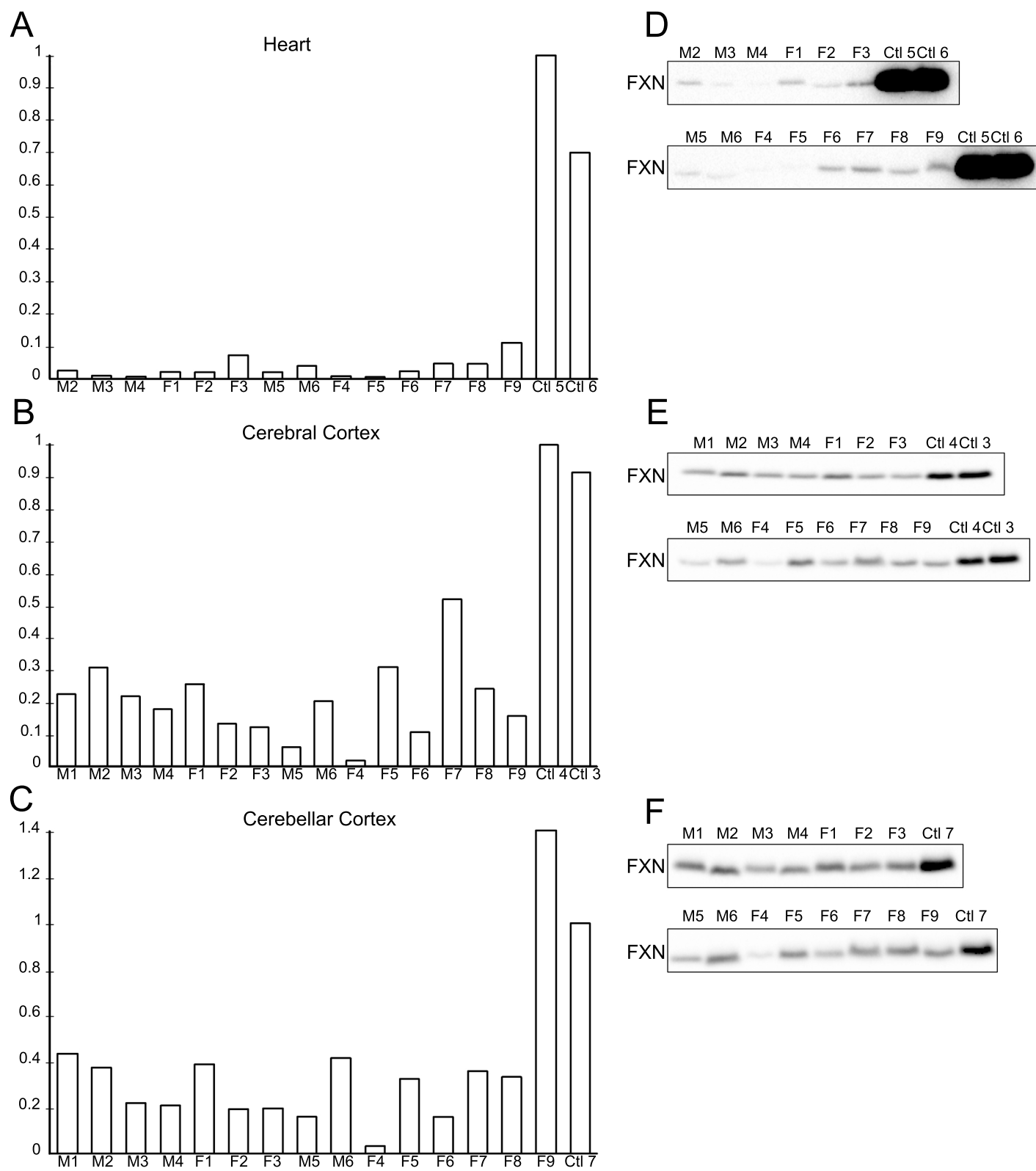

Supplement: S1 Fig — Western blots probed for FXN were performed on (A,D) heart, (B,E) cerebral cortex, and (C,F) cerebellar cortex tissues. Frataxin expression values were normalized using the mitochondrial HSP60 protein. Patient F9 exhibited very low HSP60 expression in cerebellar cortex tissue, therefore GAPDH was used for normalization. (PDF) [file pone.0189990.s001.pdf]

S2 Fig. Long, et al.

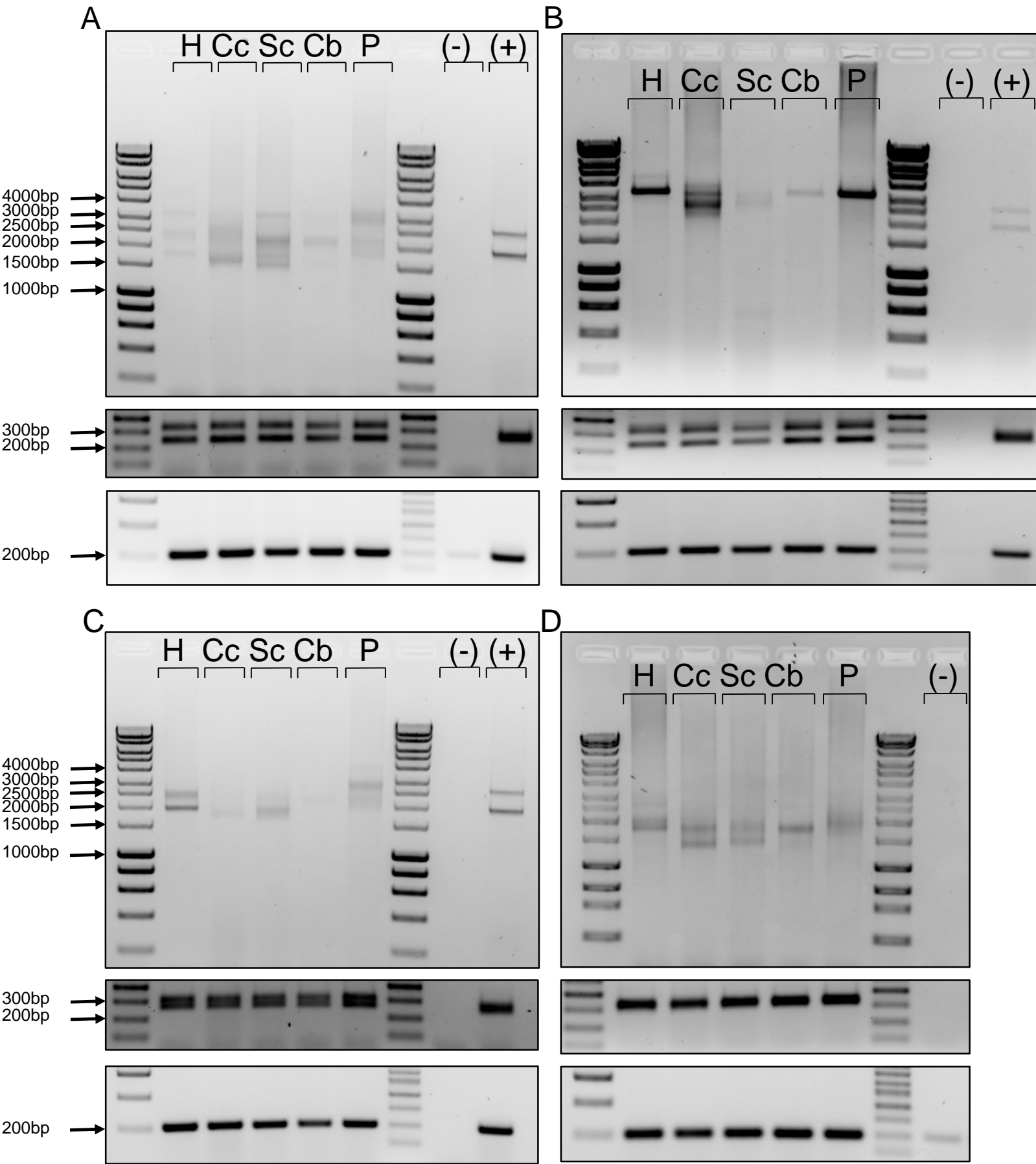

S2 Fig. Long, et al.

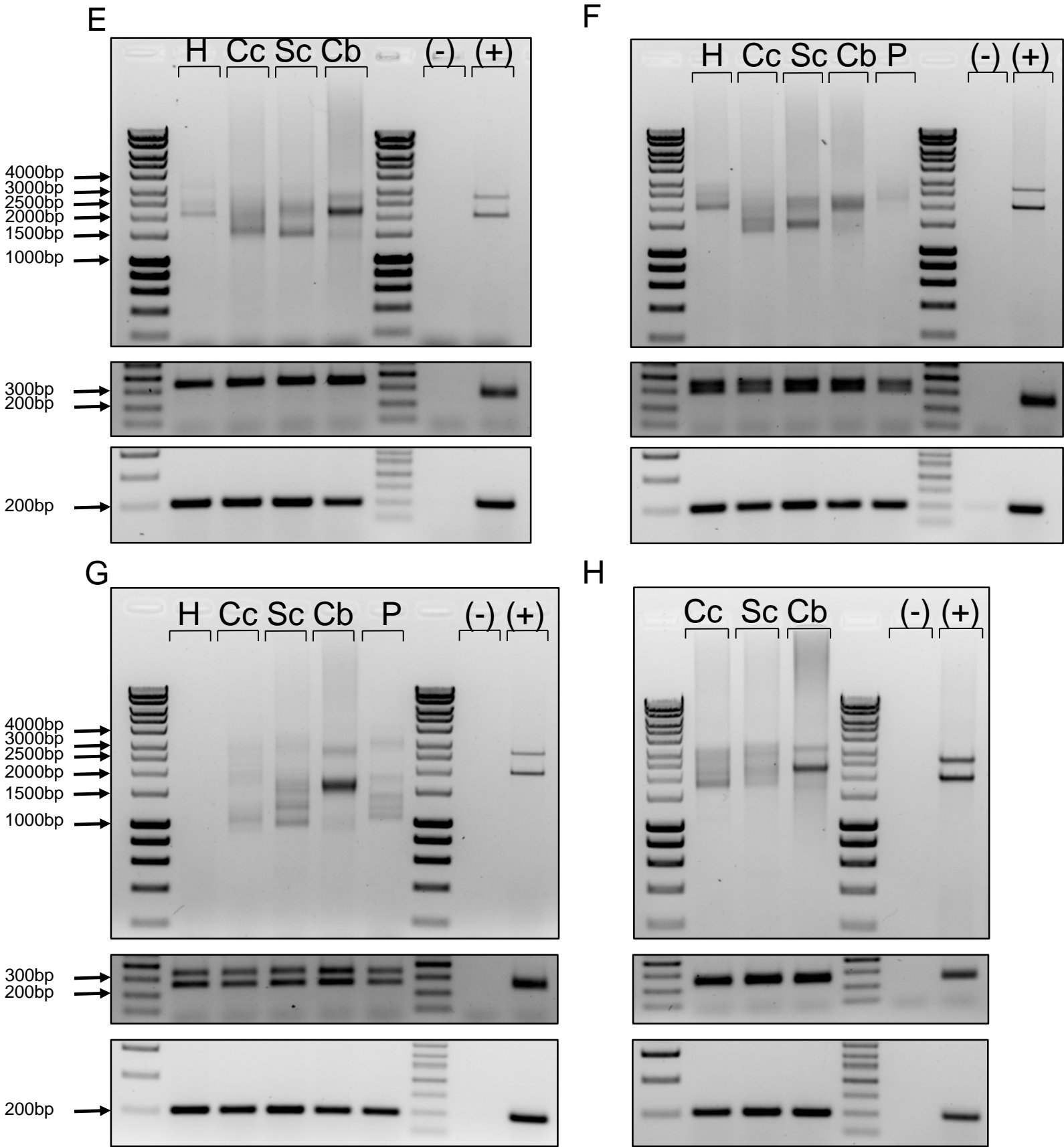

S2 Fig. Long, et al.

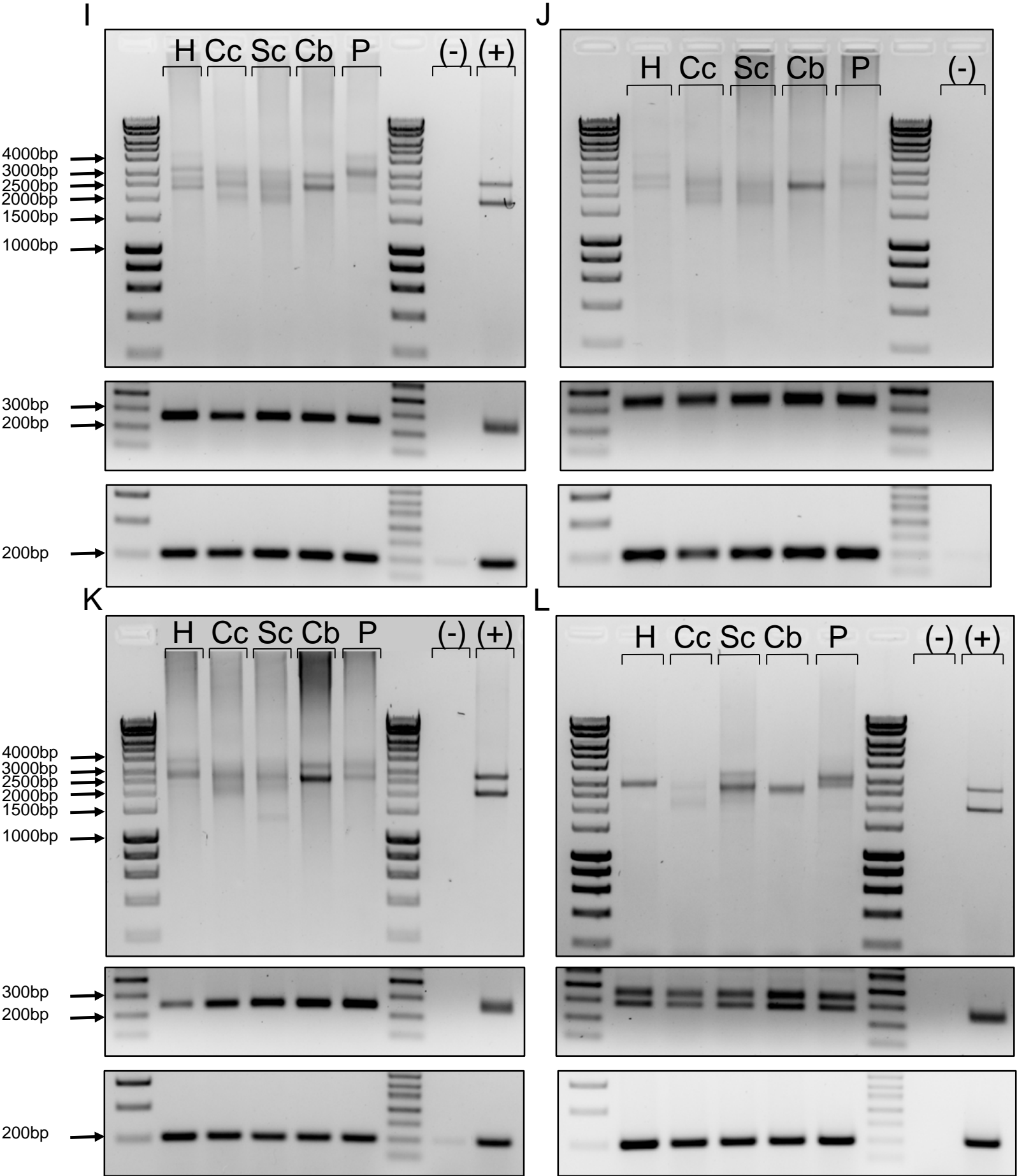

Supplement: S2 Fig — Genomic DNA was extracted from heart (H), cerebral cortex (Cc), spinal cord (Sc), cerebellar cortex (Cb) and pancreas (P) tissues. Results of PCR analyses of GAA repeat length in FRDA patient tissues (top panel). Analyses of GAA repeat instability in the 5q23 locus in patient tissues (middle panel). Amplification of an intron 1—exon 2 fragment of the FXN gene downstream of the GAA tract as control for genomic DNA quality (bottom panel). (-) represents no-template control and (+) represents positive control (genomic DNA isolated from FRDA fibroblasts). (A) F1, (B) F4, (C) F5, (D) F3, (E) F6, (F) F8, (G) F9, (H) M1, (I) M2, (J) M3, (K) M5, and (L) M4 as described in Table 1. (PDF) [file pone.0189990.s002.pdf]

S3 Fig. Long et al.

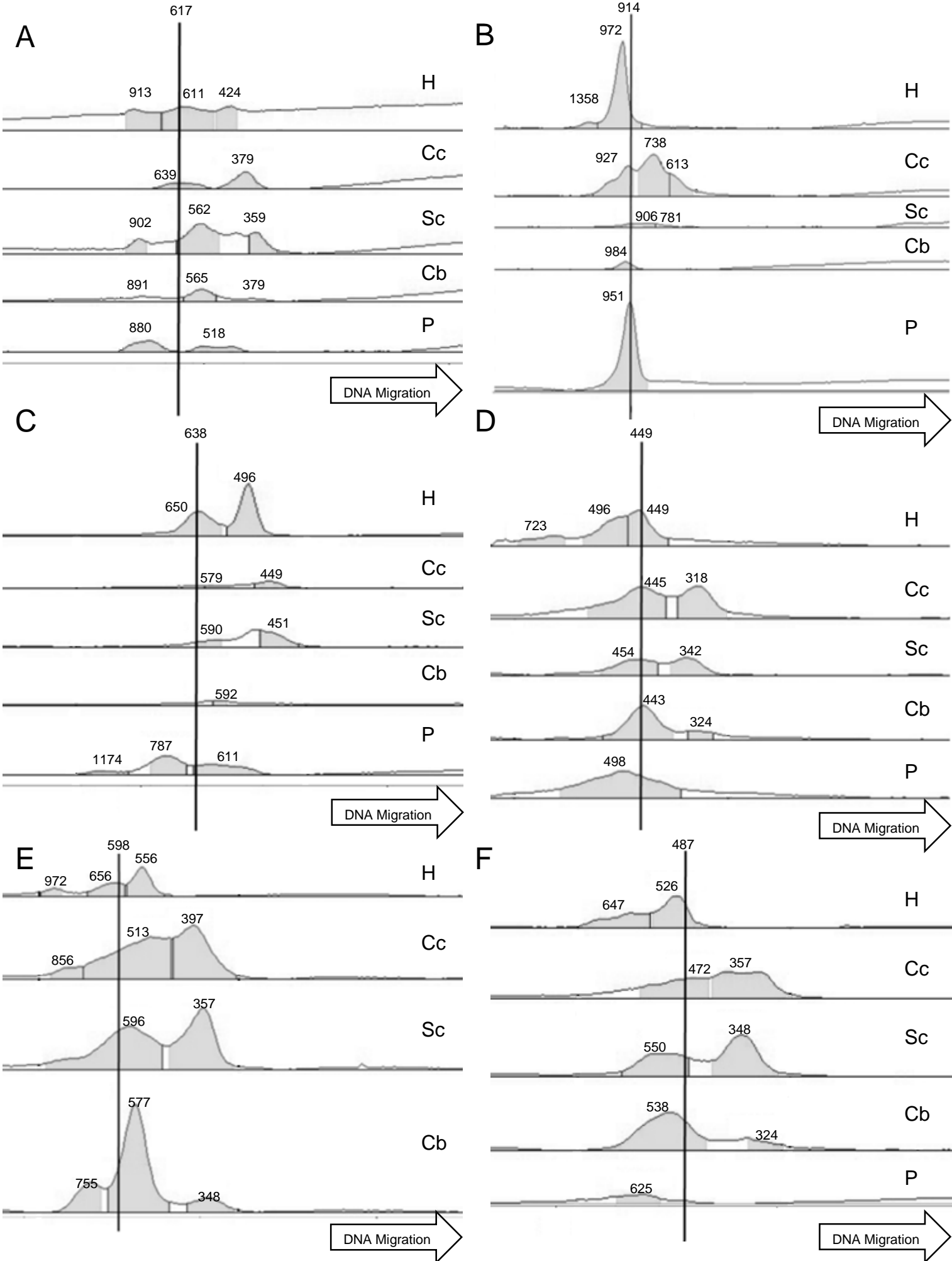

**S3 Fig. Long et al.**

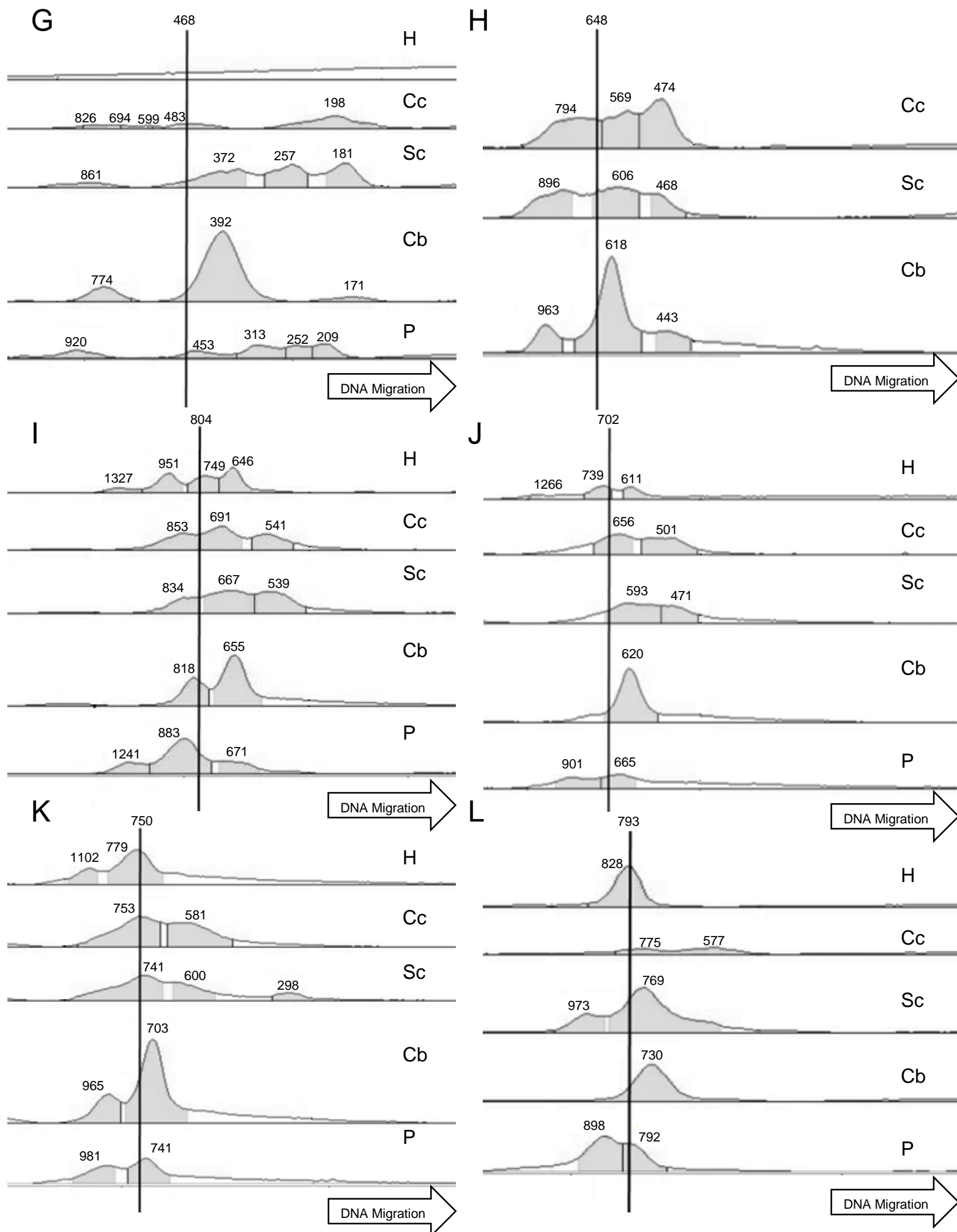

Supplement: S3 Fig — The expanded GAA repeats in the FXN gene were amplified from genomic DNA extracted from heart (H), cerebral cortex (Cc), spinal cord (Sc), cerebellar cortex (Cb) and pancreas (P) tissues isolated from FRDA patients. The band intensity of the PCR products along with the repeat sizes are shown. Solid vertical lines represent the mean of GAA repeat sizes detected across all tissues analyzed. (A) F1, (B) F4, (C) F5, (D) F3, (E) F6, (F) F8, (G) F9, (H) M1, (I) M2, (J) M3, (K) M5, and (L) M4 as described in Table 1. (PDF) [file pone.0189990.s003.pdf]

**S4 Fig. Long et al.**

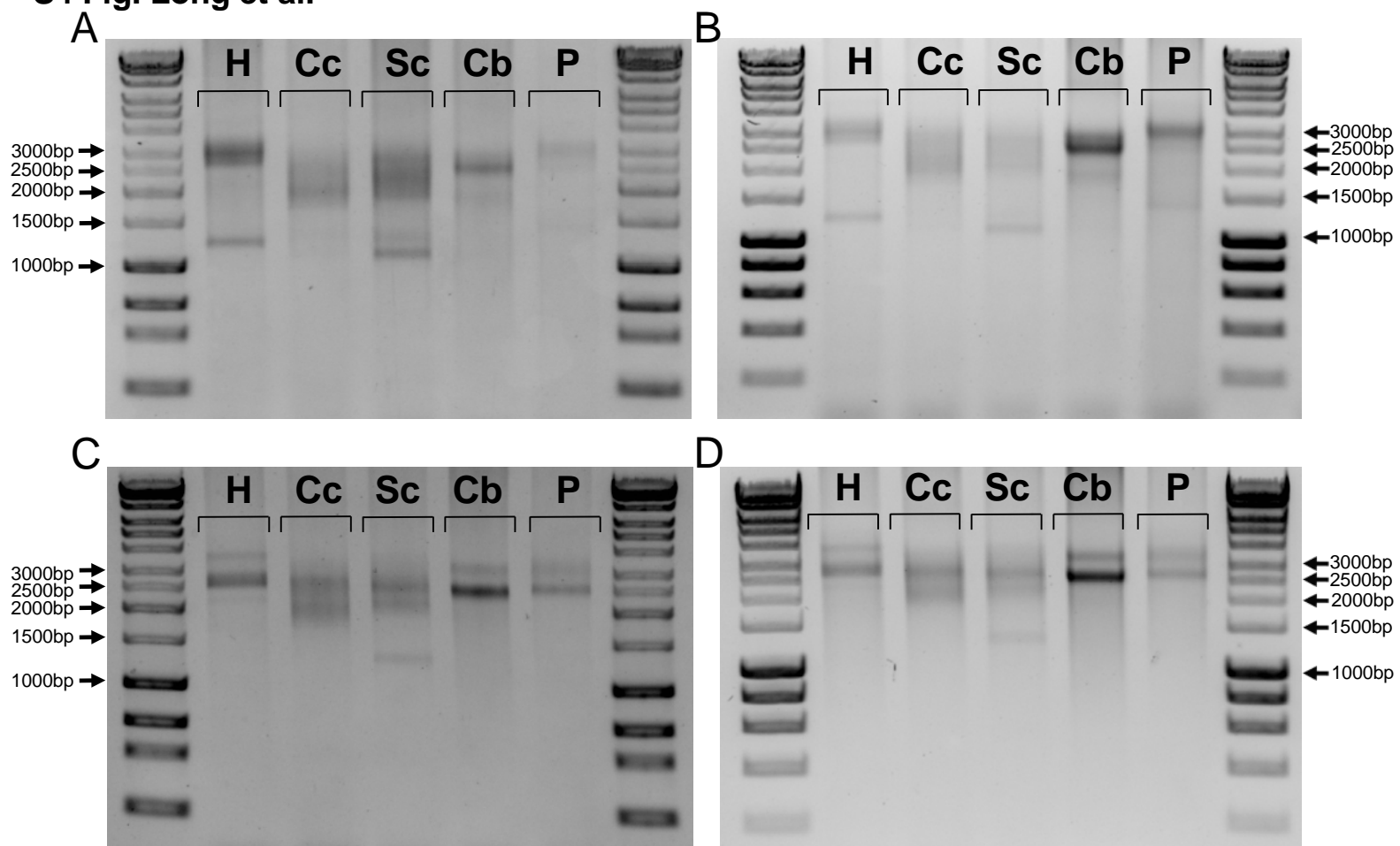

Supplement: S4 Fig — The expanded GAA repeats in the FXN gene were amplified from genomic DNA extracted from heart (H), cerebral cortex (Cc), spinal cord (Sc), cerebellar cortex (Cb) and pancreas (P) tissues isolated from FRDA patients in two independent reactions. Analyses of GAA repeat instability in tissues of FRDA patient F2; (A) experiment 1, (B) experiment 2 and in tissues of FRDA patient M5 (C) experiment 1, (D) experiment 2. Sizes of the individual PCR products were calculated for each tissue and experiment. No significant differences were observed between sizes of the GAA tracts determined in two independent experiments (t-test, p > 0.05). (PDF) [file pone.0189990.s004.pdf]

S5 Fig. Long et al.

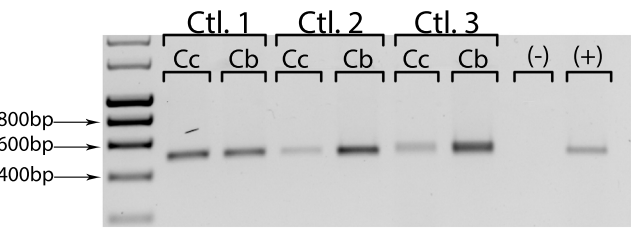

Supplement: S5 Fig — PCR analysis of the GAA repeat region in the FXN gene using genomic DNA extracted from the cerebral cortex (Cc) and cerebellar cortex (Cb) tissues of unaffected individuals. (-) represents no-template control and (+) represents positive control (genomic DNA isolated from control fibroblasts). (PDF) [file pone.0189990.s005.pdf]
